# Case report: Early molecular confirmation and sodium polystyrene sulfonate management of systemic pseudohypoaldosteronism type I

**DOI:** 10.3389/fendo.2023.1297335

**Published:** 2024-01-15

**Authors:** Einas H. Alkhatib, Deirdre Bartlett, Roopa Kanakatti Shankar, Debra Regier, Nadia Merchant

**Affiliations:** ^1^ Department of Endocrinology, Children’s National Hospital, Washington, D.C., United States; ^2^ Department of Nephrology, Lurie Children’s Hospital, Chicago, IL, United States; ^3^ Department of Pediatrics, The George Washington University School of Medicine and Health Sciences, Washington, D.C., United States; ^4^ Department of Genetics and Metabolism, Children’s National Hospital, Washington, D.C., United States

**Keywords:** pseudohypoaldoaldosteronism, sodium polystyrene sulfonate, genetics, pediatric endocrinology, kayexalate, decantation

## Abstract

**Introduction:**

Type 1 pseudohypoaldosteronism (PHA) consists of resistance to aldosterone. Neonatal presentation is characterized by salt wasting, hyperkalemia, and metabolic acidosis with high risk of mortality. Type 1 PHA can be autosomal dominant (renal type 1) or autosomal recessive (systemic type 1). Renal PHA type 1 can be feasibly managed with salt supplementation; however, systemic PHA type 1 tends to have more severe electrolyte imbalance and can be more refractory to treatment.

**Case Presentation:**

We present a case of a 3-year-old girl with systemic PHA type 1, diagnosed and confirmed molecularly in infancy, who has been successfully managed with sodium polystyrene sulfonate decanted into feeds along with sodium supplementation. On day 5 of life, a full-term female infant presented to the ED for 2 days of non-bloody, non-bilious emesis, along with hypothermia to 94°F. Laboratory results were notable for hyponatremia (Na) of 127, hyperkalemia (K) of 7.9, and acidosis with bicarbonate level of 11.2. Genetic testing ordered within a week of life confirmed PHA type 1 with a homozygous pathogenic frameshift variant in *SCNN1A* c.575delA (p.Arg192GlyfsX57). Sodium polystyrene sulfonate and feeds were decanted until the age of 16 months, and she was also continued on NaCl supplementation. She was gradually transitioned to directly administered sodium polystyrene sulfonate without any electrolyte issues. She has overall done well after gastrostomy-tube (G-tube) placement without severe hyperkalemia even with several hospitalizations for gastrointestinal or respiratory illnesses.

**Discussion/Conclusion:**

A treatment approach to systemic PHA and sodium polystyrene sulfonate administration in neonates and infants is described.

## Highlights

- Type 1 pseudohypoaldosteronism (PHA) consists of resistance to aldosterone; neonatal presentation is characterized by salt wasting, hyperkalemia, and metabolic acidosis with high risk of mortality.
- Type 1 PHA can be autosomal dominant (renal type 1) or autosomal recessive (systemic type 1); renal PHA can be feasibly managed with salt supplementation, whereas systemic PHA can be more refractory to treatment.
- Sodium polystyrene sulfonate can effectively treat hyperkalemia in type 1 PHA when it is safely decanted into feeds.
- Early molecular confirmation in the NICU/inpatient setting is instrumental in guiding definitive treatment approaches.

## Introduction

Type 1 pseudohypoaldosteronism (PHA) consists of resistance to aldosterone. Neonates may present with salt wasting, hyperkalemia, and metabolic acidosis with high mortality risk. Inheritance patterns include autosomal dominant (renal type 1) or autosomal recessive (systemic type 1). Renal PHA type 1 entails an absence of aldosterone binding to the mineralocorticoid receptor; renin and aldosterone levels are elevated. It can be feasibly managed with salt supplementation. However, systemic PHA type 1 tends to have more severe electrolyte imbalance and is due to mutations in the epithelial sodium channel (ENaC), potentially affecting sweat and salivary glands, lungs, kidneys, and colon (1). As this form can be more refractory to treatment, it is essential to learn from less commonly known approaches; further endocrinology guidance for sodium polystyrene sulfonate (brand name: Kayexalate, kalexate, and kionex; molecular weight 70,000 atomic mass units; concentration 15 g/60 mL) administration in neonates and infants is necessary (2).

There is limited guidance in the literature on specifically how to give sodium polystyrene sulfonate to neonates and infants and specifically safety of transitioning from decanting formula to giving it directly. Sodium polystyrene sulfonate dosing in several children between the ages of 1.75 and 3.25 years old ranged from 0.4 to 3.4 g/kg/day; however, the dosing and method of administration varied (3). Initial direct administration is not always feasible in neonates, to minimize risks such as GI bleeding (3).

We present a case of a 3-year-old female with systemic PHA type 1, diagnosed and confirmed molecularly in infancy, who has been successfully managed with sodium polystyrene sulfonate decanted into feeds along with sodium supplementation.

## Case report/case presentation

On day 5 of life, a full-term female infant presented to the ED for 2 days of non-bloody, non-bilious emesis, along with hypothermia to 94°F.

### Birth and family history

Pregnancy was complicated by maternal gestational diabetes requiring insulin. There were no complications with labor, and the infant was discharged after 2 days of mild hypoglycemia that resolved with oral feeding. Parents were consanguineous and from Afghanistan. Their first daughter passed away at 10 days of life; her medical diagnosis was unknown, but described to parents as “low sodium, high potassium, and concern for an abnormality on the top of the kidneys.” Parents have two healthy male children.

### ED course

Upon arrival to the Emergency Department (ED), this 5-day-old infant was found to be hypoxic to 70% and bradycardic to 70 beats per minute, with a mottled appearance. In the trauma bay, she received bag ventilation and was started on dextrose 5% with 0.45% normal saline (NS) intravenous (IV) fluids. Ampicillin and Gentamicin empiric antibiotics were started after obtaining blood and urine cultures. Laboratory results were notable for hyponatremia (Sodium, Na of 127 mmol/L), hyperkalemia (Potassium, K of 7.9 mmol/L), and acidosis with a bicarbonate level of 11.2 mmol/L. Owing to concern for hyperkalemia effects on electrocardiogram, she received three boluses of bicarbonate and two boluses of calcium gluconate.

### NICU course

She was transferred to the Neonatal Intensive Care Unit (NICU) for further management. Pediatric Endocrinology was consulted, and genitourinary exam was benign, described as “Slight rugation and hyperpigmentation of labia majora, without significant virilization, no clitoromegaly, no palpable gonads, and no posterior labial fusion. The anogential ratio of 0.34 is normal.” She received a 25-mg loading dose of IV hydrocortisone, followed by 5 mg q6h IV stress dosing, discontinued after 3 days. She was on NS fluids at maintenance rate. She was initiated on sodium chloride 4 mEq/kg/day in divided doses, titrated up to 18 mEq/kg/day, then gradually decreased. She was initiated on fludrocortisone 0.1 mg BID, which was discontinued following minimal response. Systemic PHA type 1 was thought to be most likely, but differential diagnoses also included CAH (STaR deficiency or P450SCC), or primary hypoaldosteronism. Several labs were sent, including cortisol (low at 5.6 mcg/dL), plasma renin (elevated to 118 ng/mL/h), aldosterone (elevated to 780 ng/dL), 17-hydroxyprogesterone (17-OHP, normal at 31 ng/dL), 17-OH pregnenolone (normal at 171 ng/dL), and urine Na (elevated to 138 mmol/L), as well as normal adrenal steroid hormones [androstenedione, dehydroepiandrosterone, deoxycorticosterone (DOC), 11-desoxycortisol, progesterone, and testosterone (total)] and genetic testing. A renal ultrasound demonstrated concern for a small left adrenal hemorrhage.

Personalized gene panel was sent on DOL 7. After 1 week, hyponatremia was resolved but hyperkalemia persisted from 6.3 to 9.2 mmol/L. She was transitioned from albuterol and furosemide to sodium polystyrene sulfonate via formula decantation. This was gradually advanced to a feeding regimen of 2 g of sodium polystyrene sulfonate for 100 mL of breastmilk or 8 g of sodium polystyrene sulfonate in 400 mL of breastmilk. At the time of discharge at 3 weeks of age, Na was 139 and K was 5.1 mmol/L. She was discharged on NaCl at 5.8 mEq/kg/day and an SPS regimen of 8 g per every 400 mL of formula.

Genetic testing confirmed PHA type 1 after 6 weeks, with a homozygous pathogenic frameshift variant in *SCNN1A* c.575delA (p.Arg192GlyfsX57). Data suggest that a detected deletion of one base pair within exon 3 creates a frameshift. This deletion interrupts the reading frame and creates a premature stop codon at position 57. This variant is predicted to cause loss of normal protein function through either protein truncation or nonsense-mediated mRNA decay, and it has been reported in association with PHA (4).

### Outpatient course

Over the years, she has been closely monitored outpatient by Pediatric Endocrinology and is currently 3.5 years old. She has overall done well, aside from several hospitalizations during transient gastrointestinal or respiratory illnesses. The patient’s multiple admissions are detailed in **Table 1**. Because of recurrent admissions, she had gastrostomy-tube (G-tube) placement at the age of 4 months. Sodium polystyrene sulfonate and feeds were decanted until the age of 16 months. Since then, sodium polystyrene sulfonate has been administered directly without decantation. She developed severe oral aversion and feeding intolerance, despite speech and feeding therapies. Most recently, her feeding regimen is Nutren Junior formula with 210 mL four times per day via G-tube with gradually increasing table food. She is on directly administered sodium polystyrene sulfonate 10 g four times a day. She is also on NaCl supplements 18 mEq (4.5 mL) five times a day. She continues with stable electrolytes overall. Parental written informed consent was obtained to publish this case.

**Table 1:** Inpatient admissions, electrolyte levels, and medication dosing.

Admission	Etiology	Initial labs (*Reference range: Na 135–146* *K 3.4–4.8 nmol/L)*	NaCl dose	Sodium polystyrene sulfonate dose	Additional comments
2/21/2020	Milk protein intolerance/bloody stools	Na 139, K 4.8	2.9 mEq q3h	8 g per 400 mL of Similac Advance/BM	
4/29/2020	Milk protein intolerance	Na 127, K 7	2 mEq q3h	2 g per 100 mL of Nutramigen	
5/14/2020	Feeding intolerance/emesis, electrolyte abnormalities	Na 128, K 8.8	4 mEq q3h	24 g per 800 mL of Elecare	G-tube placed
6/3/2020	Urinary tract infection (UTI), feeding intolerance	Na 132, K 7	2 mEq q3h	No change	Erythromycin trialed
7/22/2020	Feeding intolerance, dehydration	Na 138, K 4.9	1.5 mEq q6h	24 g per 800 mL of Neocate Jr.	
9/18/2020	Feeding intolerance, acute kidney injury (AKI)	Na 122, K 6.3, Cr 0.6	1.2 mEq q6h	No change	
10/11/2020	Feeding intolerance	Na 133, K 4.2	2.4 mEq q6h	No change	Feeds thickened with 2 tbsp. rice cereal
11/10/2020	Feeding intolerance	Na 125, K 4.4	No change	No change	
12/23/2020	COVID+, feeding intolerance	Na 140, K 3.7	No change	No change	
4/4/2021	Feeding intolerance, dehydration, electrolyte abnormalities	Na 126, K 7.5	No change	30 g per 800 mL of Neocate Jr.	
4/24/2021	Feeding intolerance, electrolyte abnormalities	Na 126, K 6.3	No change	No change	
5/5/2021	Norovirus gastroenteritis with feeding intolerance	Na 131, K 5.5	4 mEq q6h	No change	
6/23/2021	Gastroenteritis, feeding intolerance	Na 141, K 3.9	4 mEq q6h	30 g per 1,056 mL of Neocate Jr., plus 2 g TID directly in G-tube	Started to gradually give sodium polystyrene sulfonate directly
8/14/2021	Viral upper respiratory infection (URI), feeding intolerance	Na 137, K 3.8	8 mEq q6h	30 g per 1,056 mL of Neocate Jr., plus 3 g BID directly in G-tube	
1/7/2022	COVID+, feeding intolerance	Na 135, K 4.7	8 mEq q6h	No change	
5/9/2022	Gastroenteritis, feeding intolerance	Na 129, K 5.8	10 mEq x 5/day	10 g x 5 times/day	No longer decanting sodium polystyrene sulfonate and formula (Vivonex)
2/24/2023	Human metapneumovirus pneumonia, feeding intolerance	Na 139, K 3.7	18 mEq x 5/day	7 g x 4 times/day	

## Discussion/conclusion

For systemic PHA, sodium requirement can range from 15 to 45 g/day, whereas only 1–3 g/day are needed in renal PHA treatment (3). Sodium polystyrene sulfonate dosing often starts at 0.4–3.4 g/kg/day but can vary considerably, as aforementioned (3). In data non-specific to systemic PHA, a starting dose of 0.5 to 1 g of sodium polystyrene sulfonate powder per 100 mL of formula/breast milk is suggested (5). Additional data non-specific to systemic PHA suggest starting at 0.3–0.6 g/mEq K regardless of formula/breast milk volume (6).

Sodium polystyrene sulfonate can reduce the bioavailable content of potassium in infant formula and/or breast milk. Its onset of action is 1–2 h. Data suggest a 24% reduction in potassium within 48 h and a resolution of hyperkalemia within 72 h (3). The sodium polystyrene sulfonate binds with potassium in exchange for sodium. After this non-ingestible resin particulate settles at the bottom, the formula/breastmilk is decanted from the top. As sodium polystyrene sulfonate can bind to other oral medications and result in decreased GI absorption, it should be administered 3–6 h before or after other medications (3). Close electrolyte monitoring is necessary, as the resin can bind with calcium, magnesium, and phosphate, and it can increase sodium in formula/breast milk (3). Changes in electrolyte content of formula can vary considerably, including a 6%–89% reduction in potassium, 86%–527% increase in sodium, and 8%–84% reduction in calcium. Studies have shown that >30% of pediatric patients experienced hypokalemia, >25% hypernatremia, and >20% hypocalcemia (5). Thus, guidelines suggest obtaining baseline electrolytes, repeating in 3–7 days, and then periodically, particularly during GI illness (5).

Nephrology literature outlines the decanting process. Although sodium polystyrene sulfonate is available as a powder and liquid, powder is preferable as it forms a more noticeable precipitate. Though the precipitate is not intended to be ingested, it is not harmful if unintentional ingestion arises; however, issues with feeding tubes may arise. A quantity of four level teaspoons equals 15 g. Nutrition input is instrumental in formula decantation. Typically, an additional 100–200 mL of total volume of formula is added per decanting agent. After stirring the formula and sodium polystyrene sulfonate together and vigorously shaking for 5 min, the mixture should be covered and refrigerated for 30–60 min. When removing from the refrigerator, it is important to not further shake or mix. Then, all but 200 mL is poured over a strainer into a second container, leaving behind precipitate. This formula should be used within 24 h (5–7). Our patient is currently on Nutren Junior formula. It is important to note that this is not a low-potassium formula, as it contains 38 mEq/L of potassium. Her feeding regimen was complicated by feeding intolerance, as well as transient, nationwide formula shortages. Although this regimen is providing adequate nutritional needs, we would recommend future consideration of renal formulas such as Nutri-Renal, which has negligible mEq of potassium (8).

In our infant with systemic PHA type 1, the family and healthcare team were concerned about giving sodium polystyrene sulfonate directly since there are limited ways to decrease elevated potassium in these infants. Hence, feeds were decanted for the first 1.5 years. Although nephrology patients may require longer-term decantation, this endocrinology patient was later able to tolerate direct medication administration through G-tube. This allowed for more solid food intake and safely simplified the inpatient formula preparation process during emergency room visits and admissions. This also helped avoid admissions with every illness since the patient received sodium polystyrene sulfonate directly as she did not tolerate formula at goal amount when she was ill. Parents were able to take her to an outpatient lab to reassure that her sodium and potassium were in the normal range.

Although sodium polystyrene sulfonate is safely tolerated when properly administered, there are several potential risks to consider. Minor side effects may include nausea, emesis, and/or loose stools. In neonates, it should be administered either rectally or through decanted feeds, rather than direct oral intake. Certain products containing sorbitol are contraindicated in neonates, particularly due to increased risk for colonic necrosis. Excessive dosing or improper dilution can lead to rectal impaction of resin. Caution is necessary in premature or low birth weight infants, due to risks of GI bleeding or colonic necrosis. Sodium polystyrene sulfonate is contraindicated altogether in patients with obstructive bowel disease or neonates with reduced GI motility. Patients with underlying renal insufficiency are at increased risk for intestinal necrosis as well. Lastly, it should be used cautiously in patients requiring sodium restriction, including cardiac disease or severe hypertension (3).

Of note, there are several alternate treatment agents in nephrology patients; however, these are not widely used in PHA. Although PHA1 involves hyperchloremic acidosis with a normal anion gap (9, 10), citric acid is not a feasible alkalinizing treatment due to concern for increased hyperkalemia (11). Similarly, furosemide and thiazide can be used for acute hyperkalemia treatment, but would not be effective long-term in PHA1 due to side effects of hyponatremia (12). Sodium bicarbonate increases extracellular pH by shifting hydrogen ions out, and then shifts potassium intracellularly; however, this should not be the sole agent used, and it was minimally effective in our patient at initial presentation (13). Patiromer is a sodium-free polymer, bound with calcium, which is released in the colon in exchange for binding potassium. It is well tolerated and may be added to formula. Side effects include constipation, diarrhea, and/or hypomagnesemia (14–16). Lastly, this case also demonstrates the importance of access to rapid genetic testing for disease confirmation. Owing to the time to genetic diagnosis of this case, the institution has been able to advocate for implementing a fast-track mechanism for in-house genetic testing to decrease time to confirm the diagnosis. Other programs have implemented rapid exome or genome programs to ensure these neonates are identified in a timely manner.

To conclude, our patient with systemic PHA was effectively managed with sodium polystyrene sulfonate powder decanted into feeds with close electrolyte monitoring at baseline and during acute illnesses. She was able to safely transition to direct SPS administration. Systemic PHA can require several treatment approaches, and consistent approaches of the administration and electrolyte monitoring in decantation of feeds with SPS can be helpful to reduce hospitalizations.

## Data availability statement

The original contributions presented in the study are included in the article/supplementary material. Further inquiries can be directed to the corresponding author.

## Ethics statement

Written informed consent was obtained from the individual(s), and minor(s)’ legal guardian/next of kin, for the publication of any potentially identifiable images or data included in this article.

## Author contributions

EA: Writing – original draft, Writing – review & editing. DB: Writing – review & editing. RKS: Writing – review & editing. DR: Writing – review & editing. NM: Supervision, Writing – review & editing.
